# Bugs and drugs: a systems biology approach to characterising the effect of moxidectin on the horse’s faecal microbiome

**DOI:** 10.1186/s42523-020-00056-2

**Published:** 2020-10-14

**Authors:** S. P. Daniels, J. Leng, J. R. Swann, C. J. Proudman

**Affiliations:** 1 School of Equine Management and Science, Royal Agricultural University, Cirencester, Gloucestershire GL9 6JS UK; 2 School of Veterinary Medicine, University of Surrey, Guildford, Surrey GU2 7TE UK; 3 Department of Metabolism, Digestion and Reproduction, Faculty of Medicine, Imperial College London, London, SW7 2AZ UK

**Keywords:** Anthelmintics, Cyathostomins, Horses, Microbiome, Microbiota, Metabolome, Multi-‘omics, Strongyles

## Abstract

**Background:**

Anthelmintic treatment is a risk factor for intestinal disease in the horse, known as colic. However the mechanisms involved in the onset of disease post anthelmintic treatment are unknown. The interaction between anthelmintic drugs and the gut microbiota may be associated with this observed increase in risk of colic. Little is known about the interaction between gut microbiota and anthelmintics and how treatment may alter microbiome function. The objectives of this study were: To characterise (1) faecal microbiota, (2) feed fermentation kinetics in vitro and (3) metabolic profiles following moxidectin administration to horses with very low (0 epg) adult strongyle burdens. Hypothesis: Moxidectin will not alter (1) faecal microbiota, (2) feed fermentation in vitro, or, (3) host metabolome.

**Results:**

Moxidectin increased the relative abundance of *Deferribacter* spp. and *Spirochaetes* spp*.* observed after 160 h in moxidectin treated horses. Reduced in vitro fibre fermentation was observed 16 h following moxidectin administration in vivo (*P =* 0.001), along with lower pH in the in vitro fermentations from the moxidectin treated group. Metabolic profiles from urine samples did not differ between the treatment groups. However metabolic profiles from in vitro fermentations differed between moxidectin and control groups 16 h after treatment (*R*^2^ = 0.69, Q^2^Y = 0.48), and within the moxidectin group between 16 h and 160 h post moxidectin treatment (*R*^2^ = 0.79, Q^2^Y = 0.77). Metabolic profiles from in vitro fermentations and fermentation kinetics both indicated altered carbohydrate metabolism following in vivo treatment with moxidectin.

**Conclusions:**

These data suggest that in horses with low parasite burdens moxidectin had a small but measurable effect on both the community structure and the function of the gut microbiome.

## Background

The hindgut of the horse is populated by bacteria that are sensitive to changes in the intestinal environment. There is currently limited understanding of the relationship and communication between intestinal parasites and the hindgut microbiome within the horse [1]. Within humans and animals there is some evidence that helminth-associated alterations of gut microbiota may lead to consequences for the host organism, both localised to the gut and systemically [2].

Anthelmintic administration, specifically macrocyclic lactones, have been identified as a risk factor for the onset of intestinal disease (colic) in horses, however the reasons behind this are unknown [3–6]. Anthelmintics remain an important part of parasite control however the interaction between parasites and the gut microbiome is potentially perturbed by anthelmintic treatment. This phenomenon was initially explored in horses by Goachet et al. [7] who reported a reduction in cellulolytic bacteria and caecal pH followed by an increase in *Lactobacilli* and *Streptococci* after treatment with moxidectin identified from bacterial counts from cannulated horses in vivo. These authors also studied the effect of ivermectin and benzimidazole on the horses’ hindgut ecosystem in the same study, finding moxidectin had the greatest effect on reducing caecal pH and cellulolytic bacterial counts compared to the other two anthelmintics. More recent studies have used bacterial community profiling of faecal microbiota in horses with cyathostomin burdens following ivermectin and benzimidazole treatments [8–10]. When benzimidazole was administered to horses there was little effect seen on the bacterial community profile or the faecal metabolome [10], this conflicts with the effects on pH and bacterial counts reported by Goachet et al. [7]. Peachey et al. [9] identified differences in bacterial community profiles between horses categorised as having high and low faecal egg counts (FEC) for cyathostomins and the effect of treatment with ivermectin. The presence of parasites (macrobiota) was found to have some effect on the bacterial community profile and the greatest effect on bacterial composition was observed in the high parasite burden group. The same authors also identified that in horses with low strongyle burdens, faecal metabolites associated with carbohydrate metabolism increased following ivermectin treatment, however this was not quantified with an evaluation of functional fermentation.

Previous studies in other mammals have reported conflicting findings on the effect of nematodes on the structure and function of the microbiome. A study in pigs identified that infection with *Trichuris suis* altered the composition of the intestinal microbiota and the metabolome of the luminal content [11]. In cattle, changes in the profile of ruminal microbiota did not alter the predicted metabolic output when using a shotgun metagenomics approach to profile the bacterial community and explore its function [12]. Thus, changes in the taxonomic profile of microbial communities do not necessarily reflect metabolomic changes or functional fermentation changes within the microbiome. Currently it is unknown whether anthelmintic alone, parasite presence, parasite death or a combination of these factors following anthelmintic treatment, are responsible for the onset of post-anthelmintic intestinal disease.

Multi-omics approaches are more likely to provide answers to microbiome questions, including the effect on normal gut function, than community profiling alone. Multi-omics studies involve employing two or more differing ‘omics approaches e.g. a combination of metagenomics, transcriptomics, proteomics and metabolomics together in the same experiment to give a more holistic view of the structure and function of the microbiome [13]. Employing bacterial community profiling using the 16S rRNA gene, together with metabolomics provides information on bacterial community structure and the metabolic activity of those bacteria by evaluating the metabolites they produce. The relationship between bacterial diversity and metabolic profile has been explored in humans [14] showing a relationship between alpha diversity of microbiota and some specific metabolite concentrations e.g. hippurate. In horses bacterial metabolites including hippurate have been detected in urine and reduced excretion has been associated with altered metabolic activity within gut microbiota [15, 16].

Functional fermentation kinetics can be evaluated by using in vitro*,* batch culture systems [17]. The technique allows feed digestibility to be evaluated by measuring fermentation and dry matter disappearance using a culture inoculated with faeces. Mathematical modelling allows the calculation of kinetic rates of fermentation, the extent to which the substrate is degraded and the total fermentation, inferred by gas produced, during incubation. Combining omics’ approaches and fermentation kinetics allows for a systemic approach to evaluating microbiome structure and metabolic function alongside functional substrate digestion as a marker of digestive function.

To the authors’ knowledge this is the first study to use a multi-omics approach to characterise functional fermentation characteristics with bacterial community profiling, metabolic pathway prediction and metabolic profiling following anthelmintic administration in horses to infer the metabolic effect of anthelmintic treatment on normal gut function. Therefore this is the first study to take a community structure and mechanistic functional approach to investigating the effects of anthelmintic on the horses’ hindgut ecosystem to identify if anthelmintic administration may increase the risk of post anthelmintic colic.

The aims of this study were three fold, to characterise (1) faecal microbiota, (2) feed fermentation kinetics in vitro and (3) metabolic profiles following moxidectin administration in horses with very low parasite burdens. We hypothesise that moxidectin treatment will not alter the faecal microbiome, metabolome nor feed fermentation kinetics.

## Results

### Microbiota profiling

Total sequences within the data set were 4,034,103, the minimum number of sequences per sample was 37,883 and the maximum was 101,512. The mean number of sequences per sample was 62,070. (±14,812.8) From this 5746 unique operational taxonomic units **(**OTUs) were identified.

**Fig. 1:**
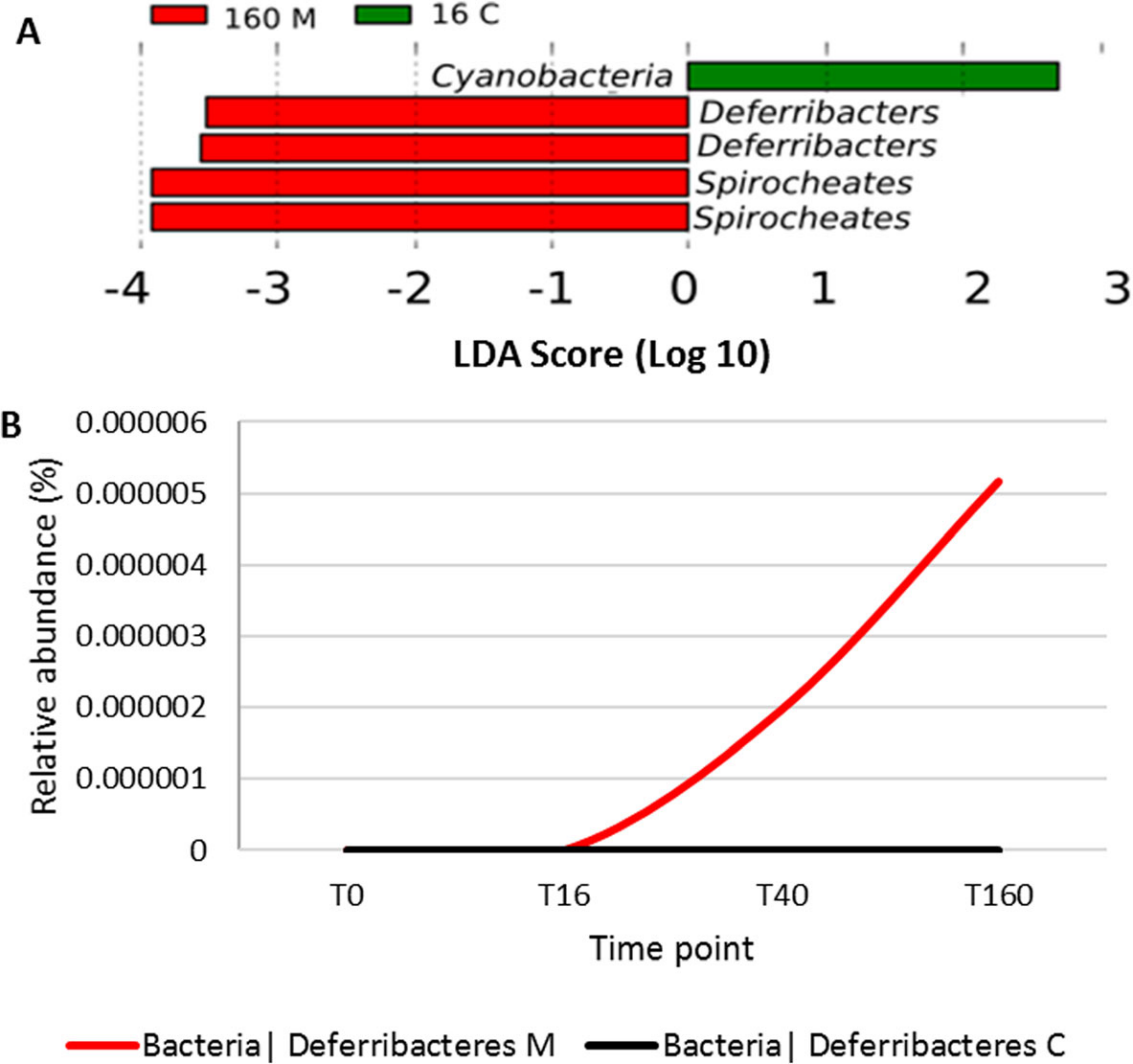
**a** LEfSe plot of OTUs differing in relative abundance between the moxidectin treated horses at 160 h post treatment (160 M) and in the control group at 16 h post treatment (16C) at genera taxonomic level. **b** Relative abundance of *Deferribacter* spp. in moxidectin (M) and control **c** groups at phyla prior to and following treatment

There were no differences in alpha nor beta diversity indices between the two treatment groups over the sampling time points (Supplementary data S1 and S2). Moxidectin treatment had a small but measurable effect on the faecal microbial community profile of the moxidectin treatment group over the sampling time points. There were moderate changes in the relative abundance of five OTUs that were identified as differing in relative abundance between the two treatment groups at genera taxonomic level 16 h following anthelmintic administration (Fig. 1a). A decrease in relative abundance of *Cyanobacteria* (MN_24) in the control group at the 16 h sampling point compared to the moxidectin treated group and control group at other time points (Supplementary data S3). An increase in relative abundance of *Deferribacters (Mucispirillum)* (Fig. 1b) and of *Spirochaetes (Treponema)* (Supplementary data S3) were observed in the moxidectin group. *Deferribacter* spp. increased in relative abundance in samples from the moxidectin group from 40 to 160 h following administration, no *Deferribacter* spp. were identified in the baseline samples. No *Deferribacter* were identified in the control group of horses at any time point (Fig. 1b). There was also an increase in relative abundance of *Spirochaetes* spp*.* in the moxidectin treated horses 160 h after moxidectin administration compared to previous time points (Supplementary data S3).

When predicting metabolic functions within the microbiome using the phylogenetic investigation of communities by reconstruction of unobserved states platform (PICRUSt) there were no differences detected between both moxidectin and control treatment groups (Supplementary data S4).

### Fermentation kinetics

**Fig. 2:**
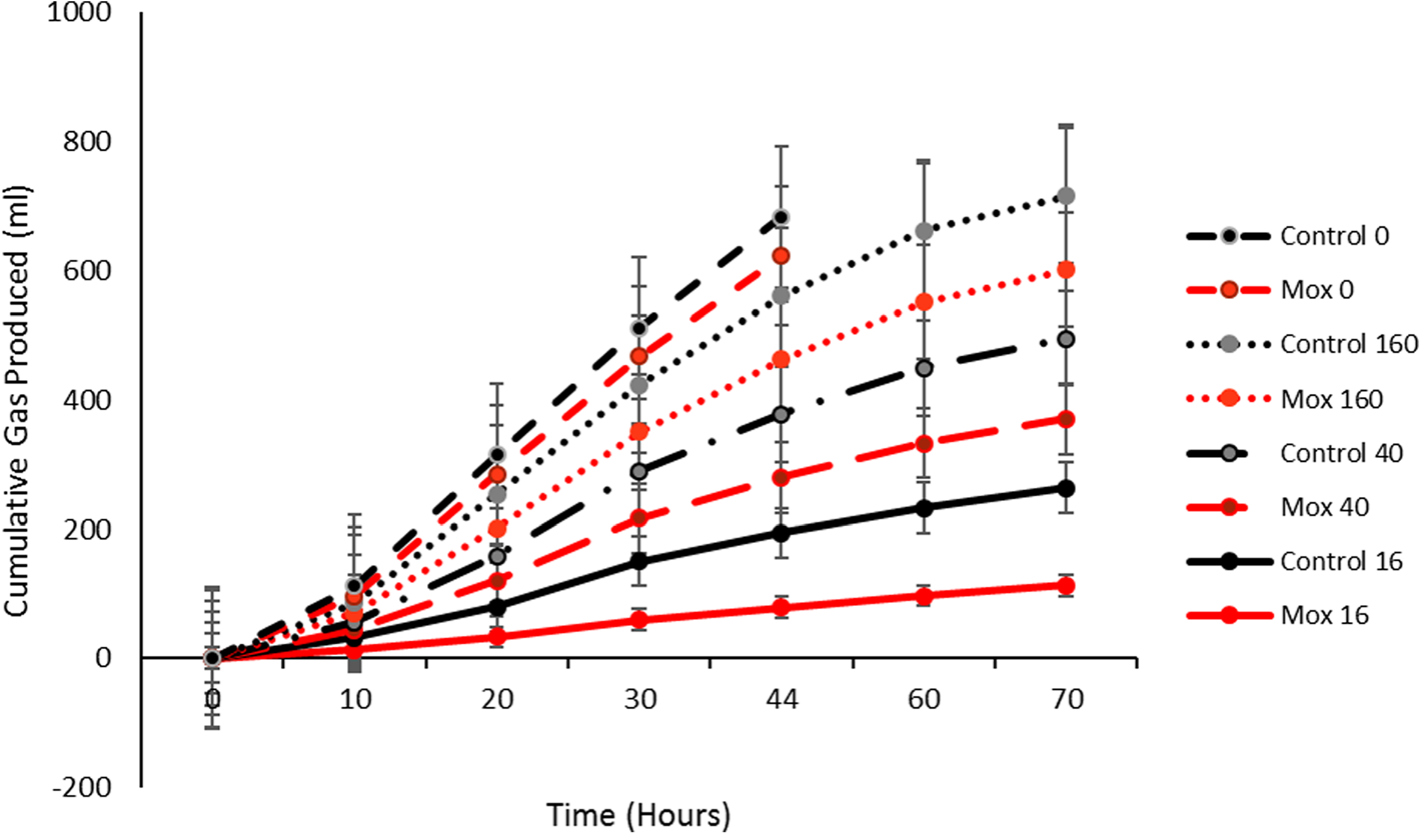
Stacked cumulative gas production curves for hay for both moxidectin and control groups prior to and 16, 40 and 160 h after moxidectin treatment. *Indicate significant differences between time points within the moxidectin group (*p =* 0.001). Error bars represent standard error

The data obtained from the fermentation experiment fitted well to the France et al. [18] model (*R*^2^ > 0.993) allowing calculation of fermentation kinetics. Differences between the treatment groups over time in cumulative fermentation profiles, whereby fermentation was significantly lower at 16 h after moxidectin administration compared to samples inoculated from non-treated controls (*P* = 0.001, Fig. 2). When considering the kinetics, the fractional rate of substrate degradation half way through fermentation and the total gas pool half way through fermentation were significantly lower in the moxidectin treated group (*P* < 0.001, Table 1) compared to the non-treated control group for the hay substrate. These changes in fermentation rate for hay were also reflected in the extent of substrate degradation which was lower in the moxidectin treated group (*P* < 0.001, Table 1).

**Table 1:** Kinetic profiles from the in vitro gas production of hay comparing the moxidectin treated group with the control group at each of the four sampling time points

Kinetics	Mox 0	Control 0	Mox 16	Control 16	Mox 40	Control 40	Mox 160	Control 160	S.E.D	Significance
Fractional Rate of Gas Production (mL/hr)	0.029 _c_	0.027 _bc_	0.016 _a_	0.023 _b_	0.026 _bc_	0.024 _bc_	0.027 _bc_	0.033 _d_	0.003219	*P* < 0.001
Gas Pool at 50% incubation (mL/g)	42.78 _a_	43.16 _a_	74.11 _c_	86.37 _d_	65.24 _bc_	71.53 _bc_	63.46 _bc_	62.15 _b_	5.529	*P* < 0.001
Extent of degradation (%)	16 _cd_	14.58_c_	7.63 _a_	10.71_b_	18.3 _d_	15.89 _cd_	14.57_c_	16.98 _cd_	1.387	*P* < 0.001

Within rows values with differing subscripts denote differences

**Fig. 3:**
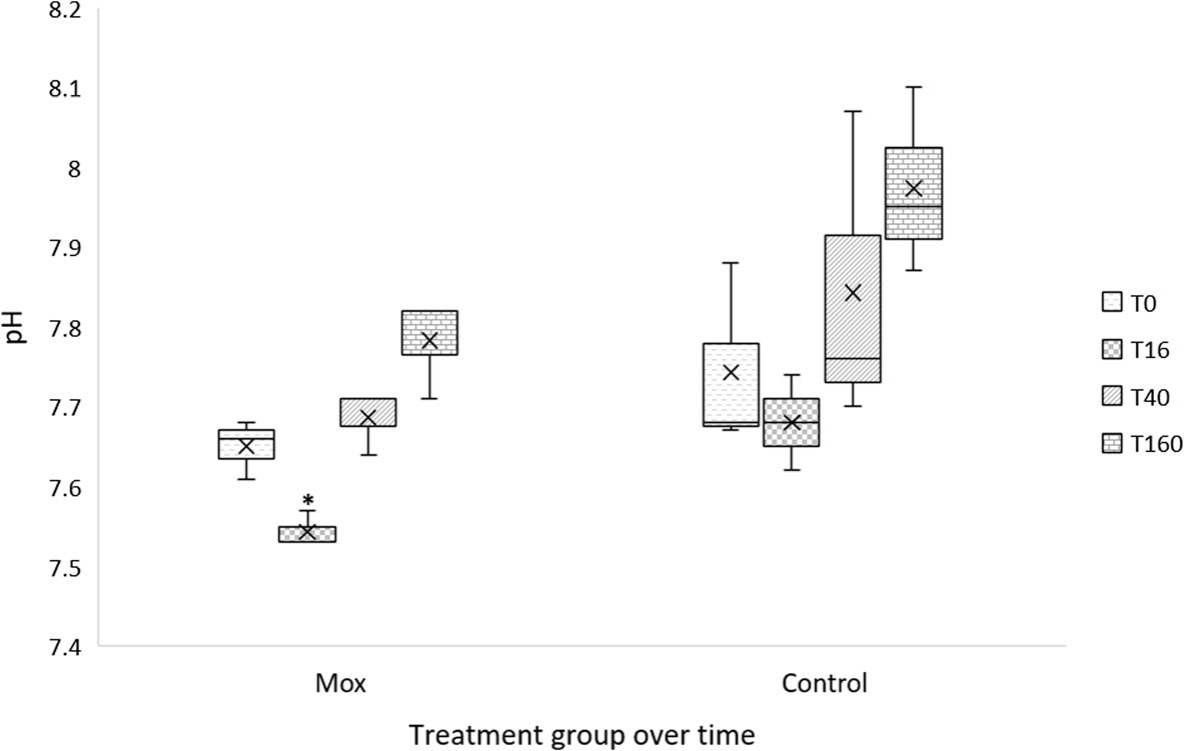
The pH of the fermentation inoculum of faeces from the horses in the moxidectin and control groups over all sampling points (0–160 h). The pH of the fermentations were significantly lower (*p* = 0.004) following inoculation with faeces from moxidectin treated horses 16 h (*) after treatment compared to controls

The pH of the fermentation inoculum differed between treatment groups and was lowest for the moxidectin group in inoculum made from faeces collected at 16 h (mean pH 7.45 ± 0.05) compared to inoculum from pre-treatment or 160 h (mean pH 7.6 ± 0.05) (*P =* 0.004) (Fig. 3).

### Metabolic phenotypes

Principal component analysis (PCA) and orthogonal projections to latent structures discriminate analysis (OPLS-DA) models built with the metabolic profiles gained from the urine samples did not identify any metabolic differences between the moxidectin and control treatment groups over the four time points (*R*^2^ = 0.3 and Q^2^Y = − 0.22, supplementary data S5).

**Fig. 4:**
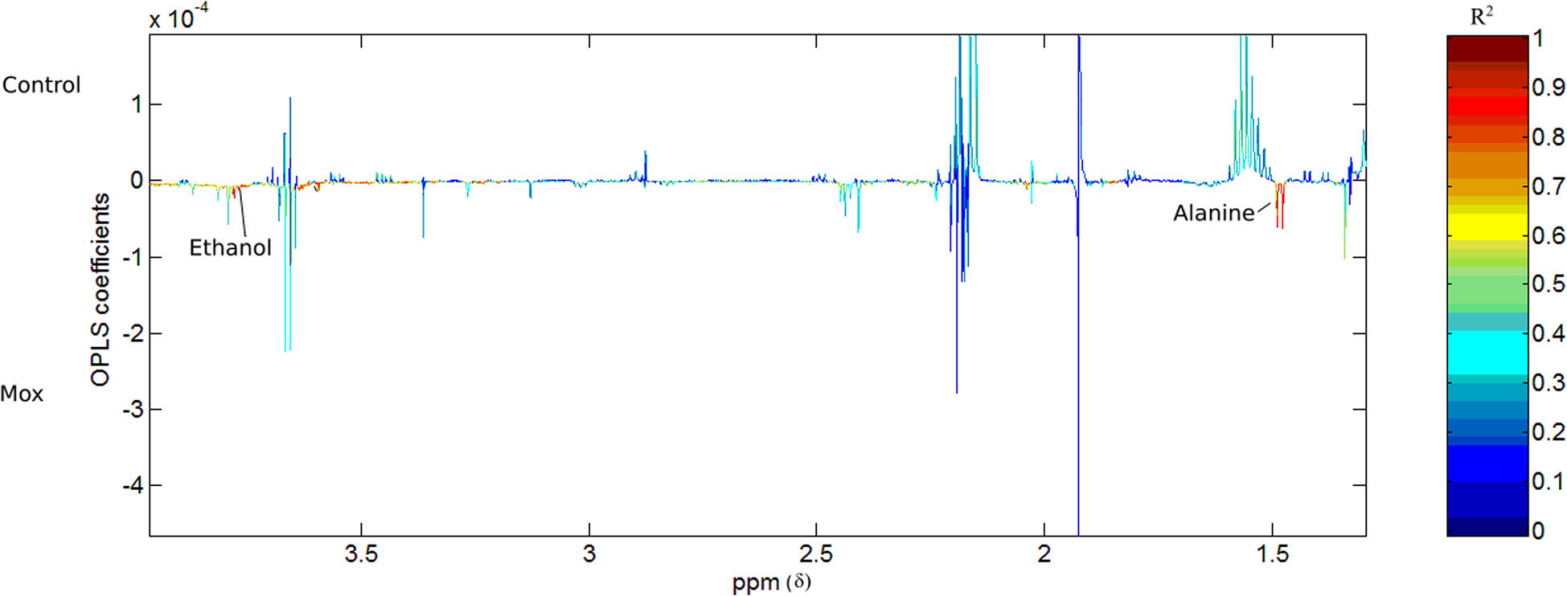
The correlation coefficient plot from the OPLS-DA model built with the metabolic profiles of samples from the fermentations of faeces collected 16 h after moxidectin administration (*R*^2^ = 0.69, Q^2^Y = 0.48)

**Fig. 5:**
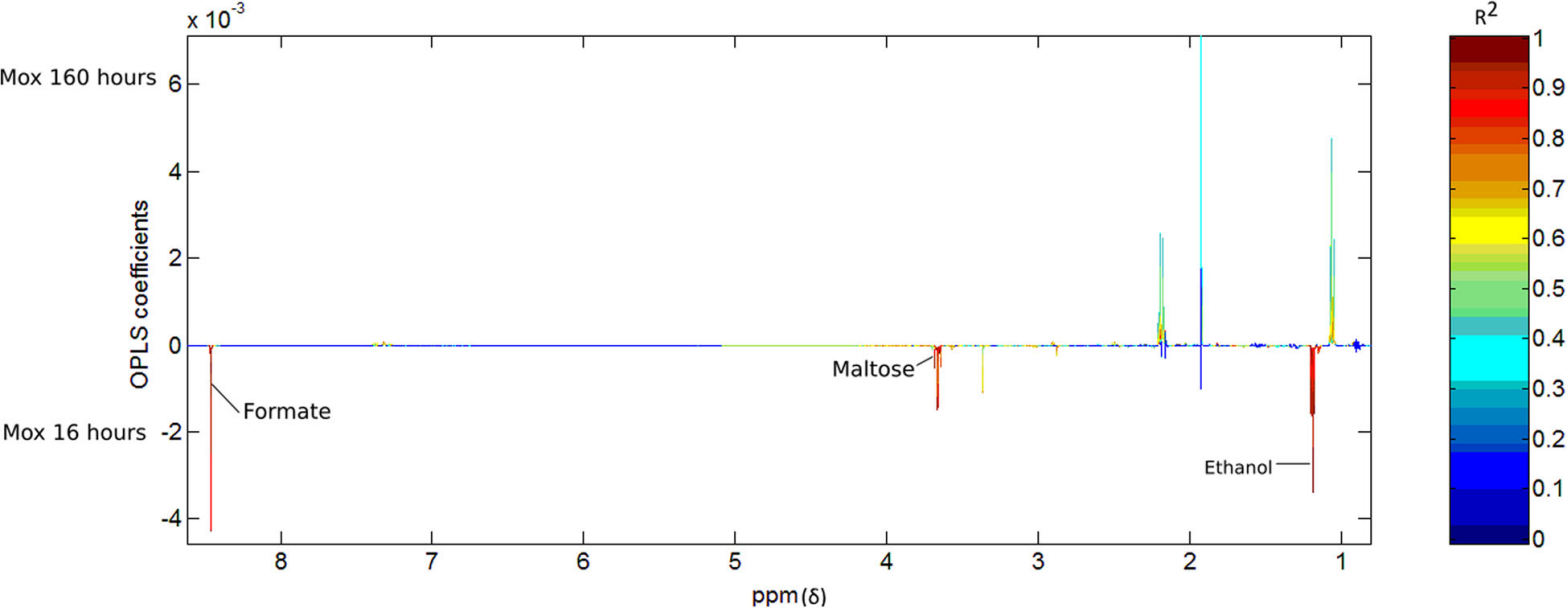
Correlation coefficient plot from the OPLS-DA model built with data from the fermentation inoculums of faeces collected at 16 h and 160 h from the horses treated with moxidectin (*R*^2^ = 0.79, Q^2^Y = 0.77)

Differences were present in the metabolic profiles of the fermentation samples following the fermentation of hay between the moxidectin group and control group 16 h after moxidectin administration (*R*^2^ = 0.69, Q^2^Y = 0.48; Fig. 5). When these metabolic differences were explored, samples from the moxidectin treatment fermentations had higher amounts of alanine and ethanol (Fig. 4). When PCA and OPLS-DA models were built with samples from the control fermentations comparing metabolite profiles within treatment groups there were no metabolic differences identified between any of the time points (*R*^2^ = 0.8, Q^2^Y = − 0.1). The OPLS-DA model built with metabolic profiles from samples from the fermentation of hay in microbial inoculum from the moxidectin treatment group identified differences between metabolite profiles between 16 h and 160 h after moxidectin administration, (*R*^2^ = 0.79, Q^2^Y = 0.77; Fig. 5). Formate, maltose and ethanol were present in greater amounts in samples from fermentations inoculated with faecal samples collected 16 h after moxidectin treatment compared to fermentations from the same animals collected 160 h after moxidectin (Fig. 5). The moxidectin treatment group had higher relative abundance of acetate, butyrate and propionate present in the fermentation samples inoculated with faeces from 160 h after treatment. Details of metabolites and their predicted concentration can be found in Table 2.

**Table 2:** Summary of OPLS-DA models, metabolites, resonances, concentrations and functions. Resonance key s; singlet, d; doublet, t; triplet, q; quartet. Metabolites referenced to Human Metabolome Database (HMDB)

	Q^**2**^Y & Permutation ***P*** values	Metabolite	1H Resonance (δ)	Correlation coefficient (R^**2**^)	Predicted Concentration (Mean & SD) (μM)		Concentration Significance	Function & reference to Human Metabolome Database
**Fermentations 16 h mox v control**	0.48 *P =* 0.01	Alanine	1.46 (d)	−0.8	**Mox** 4.57 ± 0.82	**Control** 5.14 ± 0.15	*P =* 0.4	Endogenous metabolite. Involved in urea cycle, Glucose-alanine cycle & Glycine & serine metabolism HMDB00161
		Ethanol	3.65 (q)	−0.7	4.24 ± 0.86	5.19 ± 0.33	*P =* 0.2	Metabolite, glucose and D-lactate metabolism HMDB0000108
**Fermentations mox 16 h v 160 h**	0.77 *P =* 0.01	Formate	8.46 (s)	− 0.95	**16 h** 16.38 ± 14.08	**160 h** 5.42 ± 8.64	*P =* 0.31	Microbial metabolite. Acetate & Folate metabolism. Responsible for metabolic acidosis HMDB0000142
		Ethanol	1.2 (t)	−0.95	3.05 ± 0.59	1.87 ± 1.45	*P =* 0.2	Metabolite, glucose and D-lactate metabolism HMDB0000108
		Maltose	3.41 (t)	−0.85	1.14 ± 0.22	0.7 ± 0.54	*P =* 0.25	Endogenous metabolite. Starch & sucrose metabolism HMDB00163

## Discussion

The complexity of the relationship between macrobiota, microbiota and the mammalian host is an area that has recently received attention in the literature [19, 20] and more recently specifically in the horse [21] but remains poorly understood. Here we report that moxidectin treatment did moderately alter bacterial community profile and functional fermentation kinetics temporarily following treatment. The effects of these fermentation changes were also identified in the metabolic profiles of hay fermentation culture.

Moxidectin had a small effect on the taxonomic community profile of faecal microbiota in our study; four OTUs were identified as being different in relative abundance in the faeces of horses dosed with moxidectin, however the effect on the *spirochaetes* spp. appears also to be influenced by time as a similar pattern is seen in the control group but at lower relative abundance. Kunz et al. [22] observed no changes at all in faecal microbiota profile following moxidectin and praziquantel administration, in contrast we observed some moderate taxonomic changes in our study. The sampling time points used by Kunz et al. [22] differed to those in our study, our changes in faecal microbiota were seen between 40 and 160 h after moxidectin treatment. This exceeded the time points measured by Kunz et al. [22] which only extended to 2 days post dosing, this is one possible explanation for the differences in results between studies. Peachey et al. [8, 9] reported that parasitism had a greater effect on microbiota composition than anthelmintic administration. In their study ivermectin was administered to groups of horses with low and higher FECs and they observed changes in OTUs over 14 days. No changes in alpha diversity were observed following anthelmintic treatment, a finding supported by our study.

We observed a small increase in *Deferribacter* spp. (Fig. 1b) between 40 and 160 h after moxidectin treatment. In other vertebrates increases in *Deferribacter* spp. have been observed associated with parasitism in the caecum and colon [1, 23]. *Deferribacter* spp. were not present in any of the control horse samples nor the moxidectin treated group at baseline. The increase in relative abundance of *Deferribacteres* was very small, however the horses used in our study had very low parasite burdens. In this instance any biological effect of the increase in relative abundance observed may have been negligible within the microbiome. It would be interesting to profile the relative abundance of this phyla in horses with moderate to high strongle burdens following moxidectin treatment, to see if there were a greater effect. While all of the horses in this study had no eggs seen in pre-screening FECs it is likely that they would have harbored pre-patent infections. A limitation of this study was a lack of a diagnostic to identify pre-patent infection which was not available at the time of this study. Pre-patent infection was evident from the visible excretion of cyathostomins from one of the horses the day following treatment.

*Deferribacter* spp. have previously been associated with inflammation in the gut and detected in humans and mice prior to the onset of colitis [24]. Recently Steurer et al. [25] reported an interaction between larvicidal anthelmintic treatment, cyathostomin expulsion from the mucosa and MUC2 gene expression in goblet cells following moxidectin treatment. These data lead us to speculate that an increase in the relative abundance of *Deferribacter* spp. was related to encysted cyathostomin larvae re-emerging into the gut lumen following treatment.

**Fig. 6:**
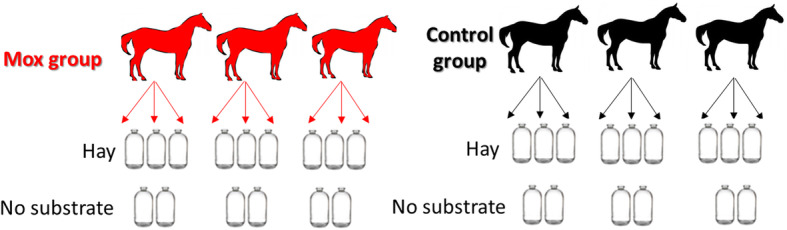
Experimental design of fermentation experiment, repeated at each sampling time point

Within the moxidectin treatment group fermentations, metabolites associated with increased acidity (Formate) were detected in greater relative abundance 16 h following treatment (Figs. 5 and 6). Metabolic profiles between the moxidectin and control treatment groups at 16 h following moxidectin differed in metabolites associated with lactate and metabolic alterations that are known to reduce pH. The metabolites that increased in relative abundance were part of the mixed acids fermentative pathway. Formate is known to increase in abundance in anaerobic environments where the pH is < 6.5. The increase in relative abundance in the metabolites detected are all associated with the fermentation of non-structural carbohydrates, this supports the findings of Peachey et al. [9] where altered carbohydrate metabolism was observed following ivermectin administration in horses with low strongyle burdens. Our metabolomic findings also support the changes in fermentation kinetics we observed 16 h following moxidectin treatment whereby the rate of fermentation, extent of substrate degradation and total substrate fermentation were reduced following moxidectin administration compared to control animals. The pH readings from faecal inoculums, made from faeces collected 16 h after moxidectin treatment, were lower than those recorded at 160 h after treatment. It is therefore plausible that in the moxidectin group, a reduction in pH in the in vitro system following treatment led to an alteration in the cellulolytic bacterial population leading to reduced fermentation. Goachet et al. [7] demonstrated a significant reduction in cellulolytic bacteria in the equid colon 1 day following moxidectin administration. These authors concluded that moxidectin administration could disturb parietal carbohydrate degradation due to the observed reduction in cellulolytic bacteria. The findings of Goachet et al. [7] support our lower fractional rate of hay degradation demonstrated in our fermentation kinetics following moxidectin treatment. However, a significant change in the cellulolytic bacterial population was not detected in vivo following moxidectin administration. Differences between fibre fermentation rates in vitro and faecal bacterial community composition of cellulolytic bacteria in vivo could be due to the closed batch culture in vitro system, or it is plausible that changes in vivo were subtle and therefore not numerically significant where considering cellulolytic OTUs but collectively the effect was great enough to alter the function in vitro. Peachey et al. [9] reported that the metabolic phenotype of horses with low cyathostomin burdens following anthelmintic treatment with ivermectin showed an increase in metabolites from carbohydrate metabolism. Our findings would support this finding in that alanine, formate, maltose and ethanol, all products of non-structural carbohydrate fermentation, were detected in increased relative abundance in the hay (structural carbohydrate) fermentation samples. Given the horses were fed a fibre-only diet and there was no starch in the fermentation system, we infer that the moxidectin, or the effect of moxidectin on any strongyle burden, altered carbohydrate metabolism in the faecal inoculum. Peachey et al. [9] concluded that anthelmintic might influence carbohydrate metabolism, detected by faecal metabolites, in horses with low parasite burdens. Our findings support this and suggest that observed changes in carbohydrate metabolism appear to have a negative effect on fibre fermentation.

When considering the pH readings from the in vitro system, all readings were > 6.2 pH and therefore unlikely to reflect major shifts in the bacterial population. The pH readings in our experiment were taken at the end of fermentation, therefore may not reflect the pH of the culture media between 10 and 50 h when differences in fermentation were observed. This is a limitation of the closed batch culture in vitro system as demonstrated by Biddle et al. [26] who identified when monitoring pH in the in vitro gas production system that the lowest pH values were recorded after 24 h of fermentation. However, by the end of fermentation (50 h) pH appeared to be buffered in the system and had returned to the baseline level. More recently the same pH effect (dropping and then buffering to near baseline) in the gas production system was observed by Moore-Colyer et al. [27]. The in vitro gas production system contains highly buffered media and thus it is likely that this played a role in pH buffering over time. Furthermore, the mean pH of the faeces used to make the initial inoculum was lower than that of the fermentation after 70 h also suggesting a buffering effect of the media. Therefore, the pH values from the fermentations in vitro may not represent true in vivo values but suggest proportional changes between groups. There was some variability within the pH values over time. The pH was lowest in the moxidectin treated horses 16 h after treatment, suggesting a causal relationship with moxidectin.

The Spirochaetes are one bacterial phyla within microbiota that play a role in acetate production. We did not observe any reductions in relative abundance of this phylum following moxidectin treatment in this group of horses in vivo, however an alteration in acetate was observed in vitro 16 h following moxidectin treatment in vivo. We did however observe an increase in Spirochaetes 160 h following moxidectin administration when acetate production appeared to return to normal. Spirochaetes only make up a small proportion of the horses hindgut microbiota in vivo. Leng et al. [28] reported that in their in vitro model they did not successfully culture Spirochaetes and that overall the relative abundance of acetate in vitro was lower than the relative abundance of acetate from in vivo samples collected from intestinal content of horses used to inoculate the model. The collectively findings of Leng et al. [28] suggest a causal relationship between this phyla and acetate production in vitro. The finding of Leng et al. [28] and the findings from our present study collectively may go some way towards explaining the reduction in fermentation following treatment.

The studies of Goachet et al. [7] were reliant on bacterial counts to identify changes in bacterial composition following anthelmintic administration. They reported differences in bacterial composition of caecal fluid from horses treated with moxidectin. Similar findings were not reported in our study. It may be that changes associated with anthelmintic administration are of small magnitude and cannot be detected from faecal samples using the techniques we employed.

The findings in our study and those reported by Peachey et al. [8, 9] suggest that the presence of cyathostomins in the hindgut ecosystem has more of an effect on microbiota composition than anthelmintic treatment. Walshe et al. [21] recently reported changes in microbiota of horses harboring cyathostomins and diagnosed pre-patent infections when dosed with moxidectin or fenbendazole. These authors conclude that an anthelmintic treatment effect on bacterial composition could not be ruled out by their study. The findings from our study, from Peachey et al. [8, 9] and Walshe et al. [21] suggest that it is macrobiota that influence microbiota composition rather than a direct anthelmintic effect.

The effects of helminths and anthelmintic treatment on gut microbiota may differ with parasite and with host species. In humans, Cooper et al. [29] reported that *Trichuris trichiura* burden and treatment with ivermectin did not alter the composition of faecal microbiota compared to untreated parasite free controls. However, alterations in the microbiota composition and the metabolome of pigs infected with *T. suis* were observed by Li et al. [11]. Further work in goats identified that *Haemonchous contortus* significantly altered the ruminal microbiota composition compared to uninfected controls [30]. A recent review by Jenkins et al. [20] suggests that in ruminants host microbiota can be found within GI tract of parasitic nematodes*.* It has been suggested that ruminal microbiota may play a role in the fitness and survival of the parasite [20]. This further supports the notion that it is the parasite rather than the anthelmintic that alters the microbiota composition.

Clark et al. [31] identified that whilst strongyle burdens did not appear to alter faecal microbiota diversity or community profile in horses, changes in microbiota diversity and OTU abundances did occur at the time of strongyle egg excretion. These authors also predicted differences in metabolic pathways around the time of strongyle egg excretion using PICRUSt. The findings of Clark et al. [31] suggest changes in microbiota and metabolic pathways within the microbiome associated with helminth presence. Peachey et al. [8] concluded that in the presence of cyathostomins (> 100 epg), alterations to bacterial community profile may alter the host immune response to allow cyathostomin survival. Recently Walshe et al. [21] reported that in horses with positive FEC (~ 400 epg) and positive encysted cyathostomin ELISA, when treated with fenbendazole or moxidectin, led to altered bacterial community profile in faeces. Bacterial composition changes returned to baseline within 14 days of treatment. The authors also reported an increase in inflammatory biomarkers. They concluded that anthelmintic treatment, leading to cyathostomin death, was most likely to have caused temporary disturbance to the hindgut ecosystem leading to changes in bacterial community profile and an increase in inflammatory biomarkers, but could not rule out the influence of anthelmintic. Taken together, the findings of Walshe et al. [21], Peachey et al. [9] and our own findings suggest that changes in microbiota associated with macrobiota may be associated with parasite death following anthelmintic treatment.

One of the inherent problems with comparing microbial profiling studies is differences in methodological approaches. Different bacterial DNA extraction methods have been used by different groups followed by different bioinformatics pipelines all of which make it difficult to directly compare findings. The findings of the aforementioned studies collectively suggest this is an area that requires further investigation.

In our study no differences in predicted metabolic pathways were observed between treatment groups when using PICRUSt. It may be that very low levels of parasitism and anthelmintic treatment do not alter metabolic pathways. It has also been reported that where there are limited differences in OTUs between groups detected by bacterial community profiling, PICRUSt is unlikely to detect changes in the predicted metabolic pathways [32]. There are also limitations in using 16S rRNA sequences to predict metabolic function, especially where structure can change but function does not alter [12]. While these predictive tools can be useful, whole genome sequencing is a more reliable method to determine metabolic pathways.

While we identified changes in the metabolic profiles using in vitro fermentations we did not observe any changes in host urinary metabolic profiles. Urine was selected as the primary biofluid for metabolomics informed by the works of Escalona et al. [15]. Escalona et al. [15] recommend urine for equid metabolomics as this biofluid yielded the greatest metabolic profiles when compared to faeces and serum. For our study design faeces may have been a more appropriate biofluid for metabonomic analysis as we were more interested in the intestinal/faecal environment.

In vitro models may not exactly reflect a microbial ecosystem in vivo but can be representative of that of the horses’ hindgut [28]. The in vitro gas production method has been identified as a suitable dynamic estimate of feed fermentation to predict the feed digestion pattern in vivo [17, 33]. While in vitro models might not provide an exact representation of the gut environment, our in vitro findings should be indicative of an in vivo response to moxidectin treatment.

In the last decade ‘omics studies have significantly increased the ability to characterize bacterial community profiles of unculturable bacteria. However bacterial composition does not necessarily reflect metabolic function [12]. Therefore charactering bacterial community presence alone does not provide meaningful functional data [34]. Metabolic profiling does provide a much greater insight into bacterial metabolism, however this can be a more powerful when using a multi-omics approach. Multi-omics approaches, provide a more holistic view of both structure and metabolic function. In ruminant studies Newbold and Ramos-Morales [34] predict a revival of batch culture work alongside ‘omics approaches to increase understanding of gut microbe structure and function. By including functional fermentation in our study and analyzing the metabolic profile of that substrate, thus taking a culture-omics approach, we have considered both in vivo data and modelled in vitro the effects of anthelmintic treatment on hindgut feed fermentation in horses. The holistic approach employed within our current study could also be used to investigate the effects of macrobiota within the gut microbiome at the time of anthelmintic administration.

There are some limitations to this study that should be recognized when interpreting the results. The feed fermentation element was conducted on a sub sample of donor horses from the main experiment (*n* = 7), this was due to practicality of sample collection, experiment set up and gas reading times. Furthermore in the pre-treatment fermentation experiment fermentation ceased earlier than the post treatment sampling time points, however the profile and kinetics of both treatment groups at this time point were comparable to the following time points. The fermentation samples that underwent metabonomic analysis were from a small sample group and this may explain the lack of difference in the predicted concentrations of the differing metabolites detected. In our study we used faeces as a proxy to represent the horses’ hindgut, however this does not necessarily reflect all regions of the hindgut [35, 36]. Faeces are commonly used as a non-invasive way to represent the hindgut ecosystem [37] and thus deemed a suitable proxy for our experiment. Notwithstanding these limitations, we believe these data do provide a meaningful insight into the effects on moxidectin treatment on the equine faecal microbiome.

Part of the rationale behind this study was to further explore anthelmintic administration as a risk factor for colic [5]. Our findings and those of others [4, 5, 9, 22] suggest that macrocyclic lactones, which have been associated with colic onset, are unlikely to be responsible for significant changes in microbiota composition. However, our findings do identify some transient functional metabolic changes that occurred following moxidectin treatment. Taken together with recent findings from other groups, our work adds further weight to the hypothesis that changes in the hindgut microbiome following anthelmintic treatment in horses may be associated with parasite death rather than with anthelmintic treatment *per se*.

## Conclusions

The findings from this study suggest that treatment of horses with very low parasite burdens with oral moxidectin results in a small but measurable effect on the microbiota composition. Temporary functional changes in the faecal microbiome were also observed. These findings warrant further investigation in horses with a larger parasite burdens, with particular focus on the effects of parasite death on the composition and metabolic function of the microbiome and its role in intestinal disease in horses.

## Methods

### Study population

A group of 17 polo ponies in Gloucestershire were recruited. Horses comprised mares (*n* = 11) and geldings (*n* = 6), mean age 12 ± 3.5 years. Breeds represented were Thoroughbreds (*n* = 4), Thoroughbred cross Argentinian polo pony (*n* = 8), Argentinian polo pony (*n* = 4) and Argentinian polo pony cross Quarter horse (*n* = 1). Horses were on pasture turnout from September 2015 – April 2016, during this time horses were not exercised and received no concentrate feeds. Grazing was supplemented with haylage ad libitum from January to April 2016.

### Parasite management

Routine parasite management for this group was identified by the owners as orally administered moxidectin (Equest, Zoetis, UK) three times annually at 0.4 mg/kg of bodyweight as recommended by the manufacturer, this regimen had remained the same over the past 3 years. Pre-intervention faecal egg counts (FEC) were conducted on all horses on three occasions during September 2015, January and February 2016 using a modified McMaster technique of Coles et al. [38] as described by Daniels and Proudman [39]. For all screening tests no nematode eggs were seen and no anthelmintic treatment was given between September 2015 and the start of the trial in March 2016. All horses were in good health and had good body condition, with no history of parasitic disease. Based upon average animal age, management regimen and FEC results all study subjects were categorised as low risk for parasitism, with presumed low adult strongyle burden.

### Study design and sampling

Sampling took place during March 2016. Horses were randomly assigned to treatment groups using a sampling function in R 3.3.2; moxidectin group (*n* = 9, seven mares and two geldings mean age 12 ± 4.5 years) and a control group (*n* = 8, five mares and three geldings mean age 13 ± 2.2 years).

From each treatment group a sub-population of horses was randomly assigned for feed fermentation kinetics, moxidectin group (*n* = 4) and control group (*n* = 3). Sample size estimates for bacterial community profiling were based upon previous profiling studies [21, 39] gave 95% power to detect 25% change in bacterial community profile between treatment groups. For the in vitro fermentations our sample gave 80% power and 95% confidence to detect a two-fold difference in fermentation kinetics between treatment groups when calculated using G*Power (Version 3.9.1.2) [40]. Sampling time points, informed by previously reported excretion times for moxidectin [41], were: prior to treatment (0), then 16, 40 and 160 h post treatment.

Free catch freshly voided faecal and urine samples were collected from all horses at all-time points. These samples were placed immediately on ice for transport to the laboratory, samples were frozen at − 80 °C within 2 h of collection. Faecal and urine samples were collected from 6 am at each of the sampling time points. Faeces for bacterial community profiling were sampled at random from the whole faecal pile avoiding faeces that had been in contact with the external environment e.g. soil. Faecal samples were collected into 50 mL plastic tubes (Fisher Scientific, Loughborough, UK). Separate faecal samples, collected at the same time, for fermentation kinetics were placed into pre-warmed thermos flasks directly after voiding. These samples were transported in an insulated container and placed in an incubator at 37 °C on arrival at the laboratory. Inoculation of the gas production system occurred within 4 h of faecal collection, flasks were placed in an incubator within 3 h of faecal voiding.

### Anthelmintic treatment

Horses in the moxidectin treatment group were dosed with moxidectin at approximately 4 pm in the afternoon of day 0 after pre-treatment samples were collected. Prior to dosing horses in the moxidectin treatment group had their weight estimated using a commercial weight tape. Due to known inaccuracies of this method [42], treatment weights were rounded up ≥50 kg. Moxidectin (Equest, Zoetis, UK) 18.92 mg/g was administered orally using the dosing device provided at as per the manufacturer’s instructions at 0.4 mg/kg of bodyweight. None of the horses demonstrated any signs of colic when observed over 24 h following moxidectin administration. Faecal consistency and feed intake also remained normal for these animals following treatment. However, one of the moxidectin treated horses was observed excreting cyathstomins the day following treatment.

### Profiling of faecal microbiota

DNA was extracted from 200 mg of each faecal sample collected and from two blank samples, where the extraction was conducted without faeces to eliminate OTU contamination from within the extraction kit. The PSP® Spin Stool DNA Kit (Stratec Molecular, Germany) was used following the manufacturer’s instructions. Pre-sequencing, aliquots of extracted DNA were amplified with universal primers for the V4 and V5 regions of the 16S rRNA gene using forward U515F (5′-GTGYCAGCMGCCGCGGTA) and reverse U927R (5′-CCCGYCAATTCMTTTRAGT) primers [43]. Amplicons were purified using 0.8 volumes of Ampure XP magnetic beads (Beckman Coulter, High Wycombe, UK). Each sample was then tagged with a unique pair of indices and the sequencing primer, using Nextera XT v2 Index kits (Illumina, Cambridge, UK), and 2x KAPA HiFi HotStart ReadyMix (Roche, UK) using the following cycling conditions: 95 °C for 3 min; 10 cycles of 95 °C for 30 s, 55 °C for 30 s, 72 °C for 30 s; followed by 72 °C for 5 min. Index-tagged amplicons were purified using 0.8 volumes of Ampure XP magnetic beads (Beckman Coulter, High Wycombe, UK). The concentration of each sample was measured using the fluorescence-based Quantifluor assay (Promega, Southampton, UK). Concentrations were normalized before pooling all samples, each of which would be subsequently identified by its unique index combination. Sequencing was performed on an Illumina MiSeq with 2 × 300 base reads according to the manufacturer’s instructions (Illumina, Cambridge. UK) by the Animal and Plant Health Agency. Bioinformatics were conducted using the QIIME pipeline (Version 1.8.0) [44]. Sequences were demultiplexed and chimeric reads filtered out. Sequences were clustered and assigned to operational taxonomic units (OTUs) of 97% similarity using USEARCH [45]. Low quality reads, those with low abundance were removed from the table. A representative sequence from each OTU cluster was aligned to the Greengenes database (Version 13.8) [46] using PyNAST [44]. FastTree [47] was then used to create a maximum likelihood phylogenetic tree. Taxonomic assignment of OTU representatives was conducted using the Ribosomal Database Project (RDP) classifier (Version 2.2) [48] informed by the Greengenes reference database. The OTU table was not rarefied [49]. Rarefaction analysis and calculation of diversity indices were conducted using QIIME, alpha diversity was calculated using Chao 1 index and Observed species index. Beta diversity was calculated using the weighted-UniFrac method [50]. The OTU table was then summarised at taxonomic levels and used for further downstream analysis. The “phylogenetic investigation of communities by reconstruction of unobserved states” (PICRUSt) platform was used to predict the metabolic pathways from the OTU table [51]. Linear discriminant analysis effect size (LEfSe) [52] was used to identify differentially abundant OTUs from the OTU tables at phyla, order and family taxonomic levels. Kyto encyclopaedia of genes and genome (KEGG) orthologs [53] from the predicted metabolic profile between treatment groups and time points were also analysed using LEfSe.

### Fermentation kinetics

To evaluate feed fermentation the in vitro gas production method was employed. This method has been widely used to simulate in vitro fermentation in horses after being validated for use with equid faeces by Lowman et al. [17]. Prior to faecal sample collection, Timothy hay was dried in a force drawn oven at 60 °C for 24 h then milled to 1 mm particles (Fritsch, Pulversette 19, Germany). One gram of hay (dry matter) was placed into pre-labelled 125 ml serum bottles as substrate for in vitro fermentation. The chemical composition of the hay can be found in Table 3. Bottle replicates were run in triplicate for each horse with two blank bottles per horse, containing no substrate (Fig. 6). In total the experiment comprised 140 culture bottles.

**Table 3:** Chemical composition of the Timothy hay used as the substrate for in vitro gas production

Component	g/kg
Dry matter	930
Crude Protein	90
Neutral Detergent Fibre	739.5
Acid Detergent Fibre	454.4
Non-Structural Carbohydrate	175.9
Fat	0.2
Ash	110
Estimated DE	7.78 Mj/Kg

Culture media were prepared as described by Theodorou et al. [54]. The mean pH of faeces, measured as described by Müller et al. [55], that were used to create the inoculum was pH 6.8 (±0.18). Faecal inoculum was prepared using a 1:5 ratio of faeces to Van Soest media while being flushed with CO_2_. The mixture was passed through a muslin cloth to collect the liquid, this was repeated separately for each donor animal. Bottles were inoculated and adjusted to ambient pressure (zero reading on the pressure transducer display) and then incubated at 39 °C as described by Lowman et al. [56].

Increase in pressure of each bottle, reflecting fermentation, were measured using the manual pressure transducer technique of Theodorou et al. [54] at 4 hourly intervals up to 74 h post inoculation. When pressure was no longer increasing the experiment was stopped and remaining contents of each bottle were filtered using pre-weighed Whatman No.1 filter papers. Samples of inoculum were taken for pH measurement and then frozen at −80 °C for metabonomic analysis. Filter papers were oven dried at 60 °C until a constant weight was reached, then weighed to calculate loss of dry matter of substrate.

Gas volume readings were corrected for pressure using linear regression bias correction to allow for any manufacturer differences in the headspace of the bottles, then any background fermentation seen in the blank bottles was subtracted from the reading [54]. A maximum likelihood program [57] was used to fit curves to the cumulative gas profiles using the France et al. [18] model. Gas readings from 0 to 74 h and kinetic parameters such as fermentation rate, extent of substrate degradation and gas pool during fermentation were calculated using the France et al. [18] model. Data were analysed using a parallel curve regression analysis and Fishers LSD post hoc test between treatments and time points to determine differences. Analysis of kinetics were via regression and Fishers LSD was used post hoc (Genstat 18th ed., VSNi).

### Metabolic profiling

Metabolic profiles were measured by ^1^H NMR spectroscopy for urine and hay fermentation samples. Samples were diluted using a phosphate buffer as described by Escalona et al. [15]. Once prepared, 500 μl of sample was pipetted into NMR tubes before loading into a 600 MHz Bruker NMR spectrometer (Bruker, Durham, UK) equipped with a cryo-probe. For each of the urine and hay fermentation samples one-dimensional ^1^H NMR spectra were acquired with water peak suppression using a standard pulse sequence. For each sample, 8 dummy scans were followed by 32 scans and collected in 64 K data point. A spectral width of 20 ppm was used for all sample types. All spectra were automatically phased, baseline corrected, and calibrated using the TSP singlet at δ 0.0 in Topspin (Bruker).

Spectra were imported in Matlab (Mathworks, 2014 edition) and analysed with in-house scripts. Spectra were initially aligned and normalized using a probabilistic quotient approach. Principal Component Analysis (PCA) was initially used to identify differences in the metabolic profiles between and within treatment groups and time points. Orthogonal Projections to Latent-Structures-Discriminate Analysis (OPLS-DA) models were constructed to identify group specific metabolic changes. Only models with a good predictive ability (Q^2^Y > 0.4) and metabolite relative abundance differences (*R*^2^ > 0.5) [58] are reported. Models also underwent permutation testing (1000 permutations) to assess the validity of the model and ensure over-fitting was not occurring [59]. The Benjamini and Hochberg [60] method for reducing false discoveries arising from multiple testing was used to identify metabolites statistically significant between groups (*P* < 0.10).

## Supplementary information

**Additional file 1: S1.** Alpha diversity indices, Chao1 and Obs all for both treatment groups over the four sampling points.

**Additional file 2: S2.** PCoA plots of weighted unifrac beta diversity between the treatment and control groups over the four sampling points. There was no difference between the treatment groups or time points (*P* >0.05).

**Additional file 3: S3.** Relative abundance of differing OTUs, Cyanobacteria and Spirochetes, between groups over the sampling time points.

**Additional file 4: S4.** Area plot of KEGG orthologs from both treatment groups over the four sampling time points, there were no differences in predicated metabolic pathways between the groups over any of these time points.

**Additional file 5: S5.** Scores plot for urine sample metabolites for both moxidectin and control groups at each of the sampling time points, treatment did not alter metabolic profile.

## Data Availability

Amplicon sequencing data have been uploaded to the European Nucleotide Archive under project ERP118335**.** Bioinformatics scripts for 16S rRNA sequences and metabolomics can be found here: https://github.com/simond83/Bio-Scripts Other data available from the corresponding author on request.

## Abbreviations

FEC: Faecal egg count
KEGG: Kyoto encyclopedia of genes and genomes
LEfSe: Linear discriminant analysis effect size
Mox: Moxidectin
NMR: Nuclear magnetic resonance
OPLS-DA: Orthogonal projections to latent structures discriminant analysis
OTU: Operational taxonomic units
PCA: Principal component analysis
PICRUSt: Phylogenetic investigation of communities by reconstruction of unobserved states
RDP: Ribosomal database project

## References

[1] Peachey LE, Jenkins TP, Cantacessi C (2017). This gut ain't big enough for the both of us. Or is it? Helminth-microbiota interactions in veterinary species. Trends Parasitol.

[2] Cortes A, Peachey LE, Jenkins TP, Scotti R, Cantacessi C. Helminths and microbes within the vertebrate gut- not all studies are created equal. Parasitology. 2019. 10.1017/S003118201900088C. 10.1017/S003118201900088X 31258097

[3] Kaenne JB, Miller R, Ross WA, Gallagher K, Marteniuk J, Rook J (1997). Risk factors for colic in the Michigan (USA) equine population. Prev Vet Med.

[4] Cohen ND, Gibbs PG, Woods AM (1999). Dietary and other management factors associated with colic in horses. J Am Vet A.

[5] Hillyer MH, Taylor FR, Proudman CJ, Edwards GB, Smith JE, French NP (2002). Case control study to identify risk factors for simple colonic obstruction and distention colic in horses. Eq Vet J.

[6] Reid SW, Mair TS, Hillyer MH, Love S (1995). Epidemiological risk factors associated with a diagnosis of clinical cyathostomiasis in the horse. Eq Vet J.

[7] Goachet AG, Ricard JM, Jacotot E, Varloud M, Julliand V. Effect of oral administration of anthelmintics on colonic microflora of horses. In: Proceedings of French Equine Veterinary Association: Pau; 2004. https://_Effet_de_l%27administration_orale_de_trois_anthelminthiques_sur_la_microflore_colique_du_cheval. Accessed 3 Sept 2020.

[8] Peachey LE, Molena RA, Jenkins TP, Di Cesare A, Traversa D, Hodgkinson, Cantacessi C (2018). The relationship between faecal egg counts and gut microbial composition in UK thoroughbreds infected by cyathostomins. Int J Parasitol.

[9] Peachey LE, Castro C, Molena RA, Jenkins TP, Griffin JL, Cantacessi C (2019). Dysbiosis associated with acute helminth infections in herbivorous youngstock – observations and implications. Sci Rep.

[10] Crotch-Harvey L, Thomas LA, Worgan HJ, Douglas JL, Gilby DE, McEwan NR (2018). The effect of administration of fenbendazole on the microbial hindgut population of the horse. J Eq Sci.

[11] Li RW, Wu S, Li W, Navarro K, Couch RD, Hill D, Urban JF (2012). Alterations in the porcine Colon microbiota induced by the gastrointestinal nematode *Trichuris suis*. Infect Immun.

[12] Taxis TM, Wolff S, Gregg SJ, Minton NO, Zhang C, Dai J, Schnabel RD, Taylor JF, Kerley MS, Pires JC, Lamberson WR, Conant GC (2015). The players may change but the game remains: network analysis of ruminal microbiomes suggest taxonomic differences mask functional similarity. Nucleic Acids Res.

[13] Pinu FR, Beale DJ, Paten AM, Kouremenos K, Swarup S, Schirra HJ, Wishart D (2019). Systems biology and multi-omics integration: viewpoints from the metabolomics research community. Metabolites..

[14] Pallister T, Jackson MA, Martin TC, Zierer J, Jennings A, Mohney RP, MacGregor A, Steves CJ, Cassidy A, Spector TD, Menni C. Hippurate as a metabolic marker of gut microbiome diversity: Modulation by diet and relationship to metabolic syndrome. Sci Rep. 7:13670. 10.1038/s41598-017-13722-4. 10.1038/s41598-017-13722-4 PMC5651863 29057986

[15] Escalona E, Leng J, Dona A, Mirrifield C, Holmes E, Proudman CJ, Swann J (2015). Dominant components of the thoroughbred metabolome characterised by 1H-NMR spectroscopy: a metabolite atlas of common biofluids. Eq Vet J.

[16] Leng J, Proudman C, Darby A, Blow F, Townsend N, Miller A, Swann J (2018). Exploration of faecal microbiota and biomarker discovery in equine grass sickness. J Proteome Res.

[17] Lowman RS, Theodorou MK, Hyslop JJ, Dhanoa MS, Cudderford D (1999). Evaluation of *in vitro* batch culture technique for estimating the in vivo digestability and digestable energy content of equine feeds using equine faeces as a source of microbial inoculum. Anim Feed Sci Technol.

[18] France J, Dhanoa MS, Theodorou MK, Lister SJ, Davies DR, Isac D (1993). A model to interpret gas accumulation profiles associated with *in vitro* degradation of ruminant feeds. J Theor Biol.

[19] Leung JM, Graham AL, Knowles SLC. Parasite-microbiota interactions with the vertebrate gut: synthesis through an ecological lens. Front Microbiol. 2018. 10.3389/fmicb.2018.00843. 10.3389/fmicb.2018.00843 PMC5960673 29867790

[20] Jenkins TP, Brindley PJ, Gasser RB, Cantacessi C (2019). Helminth microbiomes- a hidden treasure trove? Trends. Parasitol..

[21] Walshe N, Duggan V, Cabrera-Rubio R, Crispie F, Cotter P, Feehan O, Mulcahy G. Removal of adult cyathostomins alters faecal microbiota and promotes an inflammatory phenotype in horses. Int J Parasitol. 2019. 10.1016/j.ijpara.2019.02.003. 10.1016/j.ijpara.2019.02.003 30986403

[22] Kunz IGZ, Reed KJ, Metcalf JL, Hassel DM, Coleman RJ, Hess TM, Coleman SJ (2019). Equine Fecal Microbiota Changes Associated With Anthelmintic Administration. J EqVet Sci.

[23] Kreisinger J, Bastien G, Hauffe HC, Marchesi J, Perkins SE. Interactions between multiple helminths and gut microbiota in wild rodents. Philos Trans R Soc. 2015. 10.1098/rstb.2014.0295. 10.1098/rstb.2014.0295 PMC4528493 26150661

[24] Berry D, Kuzyk O, Rauch I, Heider S, Schwab C, Hainzl E, Decker T, Muller M, Strobl B, Schleper C, Urich T, Wagner M, Kenner L, Loy A. Intestinal muicrobiota signatures associated with inflammation history in mice experiencing recurring colitis. Front Microbiol. 2015. 10.3389/fmicb.2015.01408. 10.3389/fmicb.2015.01408 PMC4678223 26697002

[25] Steurer AA, Stewart JC, Barker VD, Adams AA, Nielsen MK. Cytokine and goblet cell gene expression in equine cyathostomin infection and larvicidal anthelmintic therapy. Parasite Immunol. 2020. 10.1111/PIM.12709. 10.1111/pim.12709 32145074

[26] Biddle AS, Black SJ, Blanchard JL. An *in vitro* model of the horse gut microbiome enables identification of lactate-utilizing bacteria that differentially respond to starch induction. PLoS One. 2013. 10.1371/journal.pone.0077599. 10.1371/journal.pone.0077599 PMC3788102 24098591

[27] Moore-Colyer MJS, Tuthill P, Bannister I, Daniels S. Growth rates of Thoroughbred foals and in vitro gut health parameters when fed a cereal or an all-fibre creep feed. J Equine Vet Sci. 2020;93. 10.1016/j.jevs.2020.103191. 10.1016/j.jevs.2020.103191 32972676

[28] Leng J, Walton G, Swann J, Darby A, La Ragione R, Proudman C. “Bowel on the Bench”: Proof of Concept of a Three-Stage, *In Vitro* Fermentation Model of the Equine Large Intestine. Appl Environ Microbiol. 2020. 10.1128/AEM.02093-19. 10.1128/AEM.02093-19 PMC6912081 31676474

[29] Cooper P, Walker AW, Reyes J, Chico M, Salter SJ, Vaca M, Parkhill J (2013). Patent human infections with the whipworm, Trichuris trichiura, are not associated with alterations in the faecal microbiota. PLoS One.

[30] Li RW, Li W, Sun J, Yu P, Baldwin RL, Urban JF. The effect of helminth infection on the microbial composition and structure of the caprine abomasal microbiome. Sci Rep. 2016. 10.1038/srep20606. 10.1038/srep20606 PMC4757478 26853110

[31] Clark A, Salle G, Ballan V, Reigner F, Meynadier A, Cortet J, Koch C, Riou M, Blanchard A, Mach N. Strongyle infections and gut microbiota: profiling of resistant and susceptible horses over a grazing season. Front Physiol. 2018. 10.3389/fphys.2018.00272. 10.3389/fphys.2018.00272 PMC5871743 29618989

[32] Xu Z, Malmer D, Langille MGI, Way SF, Knight R (2014). Which is more important for classifying microbial communities: whos there of what they can do?. ISME J.

[33] Brown VE, Rymer C, Agnew RE, Givens I (2002). Relationship between in vitro gas production profiles of forages and in vivo rumen fermentation patterns in beef steers fed those forages. Anim Feed Sci Technol.

[34] Newbold CJ, Ramos-Morales E (2020). Ruminal microbiome and microbial metabolome: effects of diet and ruminant host. Animal..

[35] Dougal K, de la Fuente G, Harris PA, Girdwood SE, Pinloche E, Newbold CJ (2013). Identification of a core bacterial community within the large intestine of the horse. PLoS One.

[36] Julliand V, Grimm P (2016). Horse species symposium: the microbiome of the horses hindgut: history and current knowledge. J Anim Sci.

[37] Coles GC, Bauer C, Borgsteede FHM, Geerts S, KleiT R, Taylor MA, Waller PJ (1992). World Association for the Advancement of veterinary parasitology (WAAVP) methods for the detection of anthelmintic resistance in nematodes of veterinary importance. Vet. Parasitol..

[38] Daniels SP, Proudman CJ (2016). Ovicidal efficacy of fenbendazole after treatment of horses naturally infected with cyathostomins. Vet Parasitol.

[39] Tyma JE, Epstein JL, Whitfield-Cargile CM, Cogen ND, Giguere S (2019). Investigation of the effects of omeprazole on the faecal and gastric microbiota of healthy adult horses. Am J Vet Res.

[40] Faul F, Erdfelder E, Lang A-G, Buchner A. G*Power 3: a flexible statistical power analysis program for the social, behavioural, and biomedical sciences. Behav Res Methods. 2007;39:175–91. 10.3758/bf03193146 17695343

[41] Gokbulet C, Nolan AM, McKellar QA (2001). Plasma pharmacokinetics and faecal excretion of ivermectin, doramectin and moxidectin following oral administration in horses. Eq Vet J.

[42] Ellis JM, Hollands T (1998). Accuracy of different methods of estimating the weight of horses. Vet. Rec..

[43] Ellis RJ, Bruce KD, Jenkins C, Stothard JR, Ajarova L, Mugisha L (2013). Viney ME comparison of the distal gut microbiota from people and animals in Africa. PLoS One.

[44] Caporaso JG, Kuczynski J, Stombaugh J, Bittinger K, Bushman FD, Costello EK, Fierer N, Pena AG, Goodrich JK, Gordon JI, Huttley GA, Kelley ST, Knights D, Koenig JE, Ley RE, Lozupone CA, McDonald D, Muegge BD, Pirrung M, Reeder J, Sevinsky JR, Tumbaugh PJ, Walters WA, Widmann J, Yatsunenko T, Zaneveld J, Knight R (2010). QIIME allows analysis of high-throughput community sequencing data. Nat. Methods..

[45] Edgar RC (2010). Search and clustering orders of magnitude faster than BLAST. Bioinformatics.

[46] DeSantis TZ, Hugenholtz P, Larsen N, Rojas M, Brodie EL, Keller K, Huber T, Dalevi D, Hu P, Andersen GL (2006). Greengenes, a chimera-checked 16S rRNA gene database and workbench compatible 383 with ARB. Appl Environ Microbiol.

[47] Price MN, Dehal PS, Arkin AP (2010). FastTree 2--approximately maximum-likelihood trees for large 451 alignments. PLoS One.

[48] Wang Q, Garrity GM, Tiedje JM, Cole JR. Naıve bayesian classifier for rapid assignment of rRNA sequences into the new bacterial taxonomy. Appl Environ Microbiol. 2007. 10.1128/AEM.00062-07. 10.1128/AEM.00062-07 PMC1950982 17586664

[49] McMurdie PJ, Holmes S. Waste not, want not: why rarefying microbiome data is inadmissible. PLoS Comput Biol. 2014. 10.1371/journal.pcbi.1003531. 10.1371/journal.pcbi.1003531 PMC3974642 24699258

[50] Lozupone CA, Hamady M, Kelley ST, Knight R (2007). Quantitative and qualitative beta diversity measures lead to different insights into factors that structure microbial communities. Appl Environ Microbiol.

[51] Langille MJ, Zaneveld J, Caporaso JG, McDonald D, Knights D, Reyes JA, Clemente JC, Burkepile DD, Vega Thurber RL, Knight R, Bieko RG, Huttenhower C (2013). Predictive functional profiling of microbial communities using 16S rRNA marker gene sequencing. Nat Biotechnol.

[52] Segata N, Izard J, Waldron L, Gevers D, Miropolsky L, Garrett WS, Huttenhower C (2011). Metagenomic biomarker discovery and explanation. Genome Biol.

[53] Kyto encyclopaedia of genes and genome (KEGG) orthologs https://www.genome.jp/kegg/pathway.html Accessed May 2019.

[54] Theodorou MK, Williams BA, Dhaona MS, McAllan AB, France J (1994). A simple gas production method using a pressure transducer to determine the fermentation kinetics of ruminant feeds. Anim Feed Sci Technol.

[55] Müller CE, von Rosen D, Uden P (2008). Effect of forage conservation method of microbial flora and fermentation pattern in forage and in equine colon and faeces. Livest Sci.

[56] Lowman RS, Theodorou MK, Longland AC, Cuddeford D (1996). A comparison of equine faeces or caecal digesta as sources of inoculum for *in vitro* fermentation studies using the pressure transducer technique. Anim Sci.

[57] Ross GJS (1987). MLP Maximum Likelihood Programme.

[58] Gowan AA, Downey G, Esquerre C, O'Donnell CP (2010). Preventing over-fitting in PLS calibration models of near-infrared spectroscopy data using regression coefficients. J Chemother.

[59] Benjamini Y, Hochberg Y (1995). Controlling the false discovery rate: A practical and powerful approach to multiple testing. J Roy Stat Soc.

[60] Trygg J, Holmes E, Lundstedt T (2007). Chemometrics in metabonomics. J Proteome Res.

