# Supplementary figures and images for: Bugs and drugs: a systems biology approach to characterising the effect of moxidectin on the horse’s faecal microbiome
Source: Anim Microbiome. 2020 Oct 14;2:38. doi: 10.1186/s42523-020-00056-2 (PMC7807906; doi:10.1186/s42523-020-00056-2)

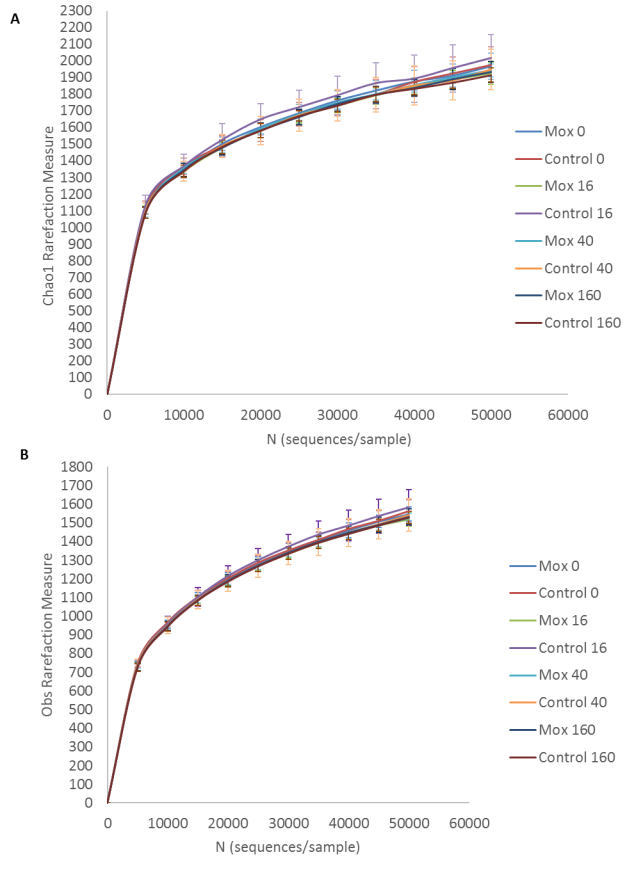

Supplement: Supplementary file 1 — Additional file 1: S1. Alpha diversity indices, Chao1 and Obs all for both treatment groups over the four sampling points. [file 42523_2020_56_MOESM1_ESM.png]

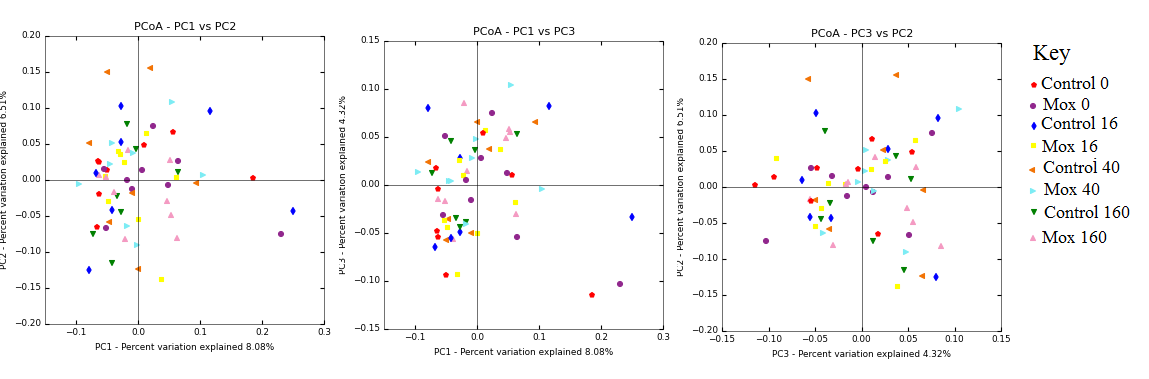

Supplement: Supplementary file 2 — Additional file 2: S2. PCoA plots of weighted unifrac beta diversity between the treatment and control groups over the four sampling points. There was no difference between the treatment groups or time points (P >0.05). [file 42523_2020_56_MOESM2_ESM.png]

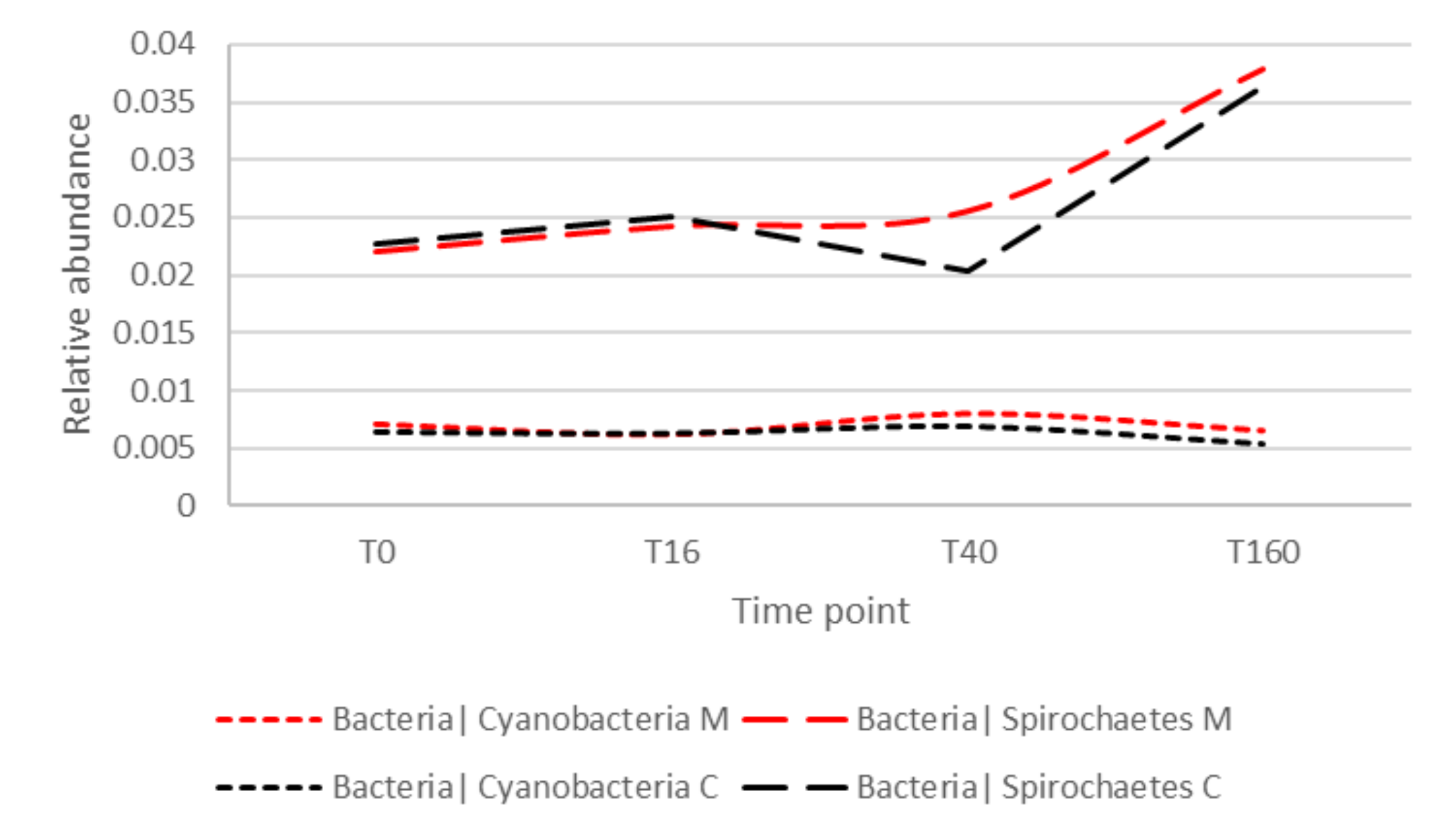

Supplement: Supplementary file 3 — Additional file 3: S3. Relative abundance of differing OTUs, Cyanobacteria and Spirochetes, between groups over the sampling time points. [file 42523_2020_56_MOESM3_ESM.png]

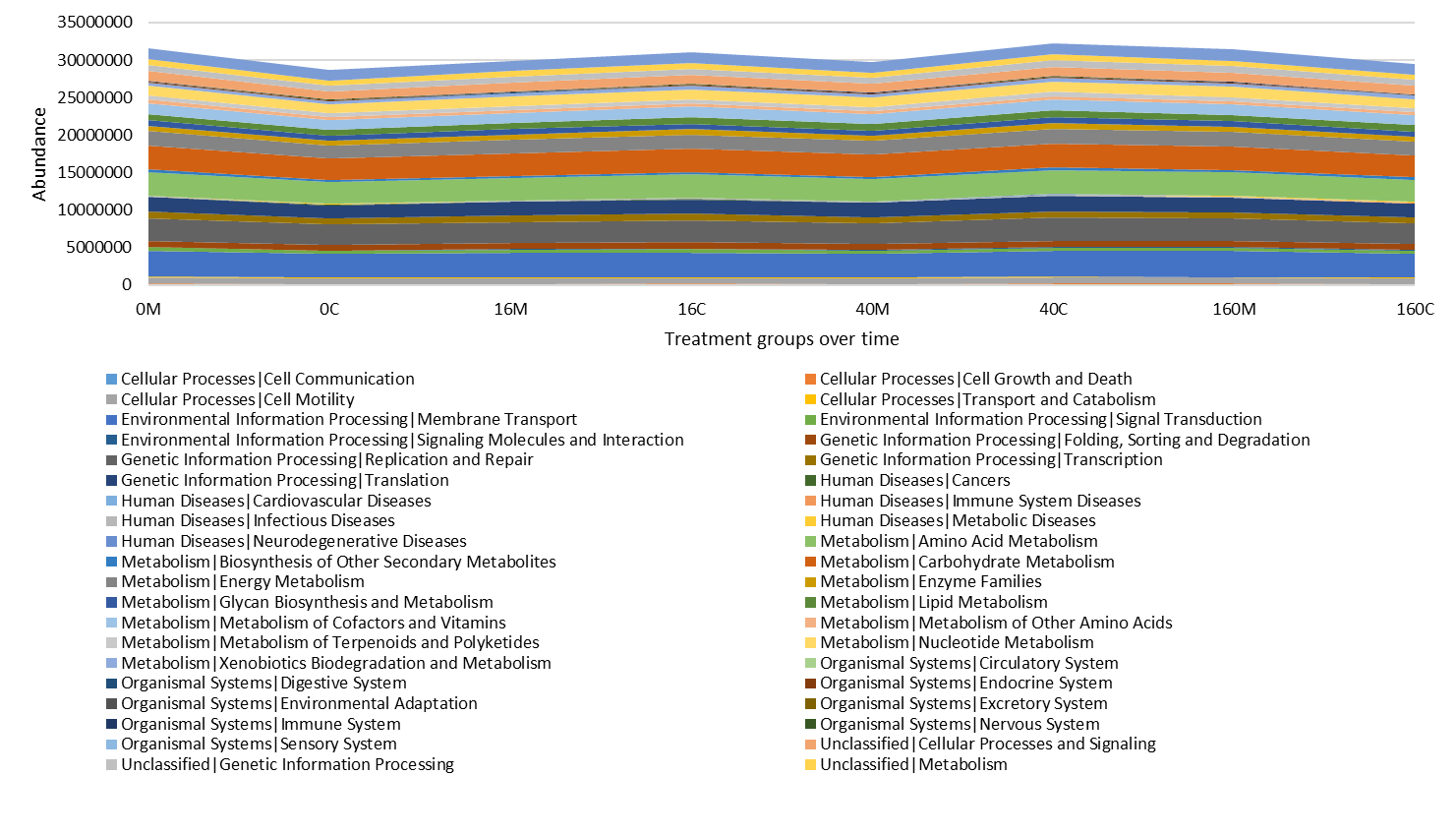

Supplement: Supplementary file 4 — Additional file 4: S4. Area plot of KEGG orthologs from both treatment groups over the four sampling time points, there were no differences in predicated metabolic pathways between the groups over any of these time points. [file 42523_2020_56_MOESM4_ESM.png]

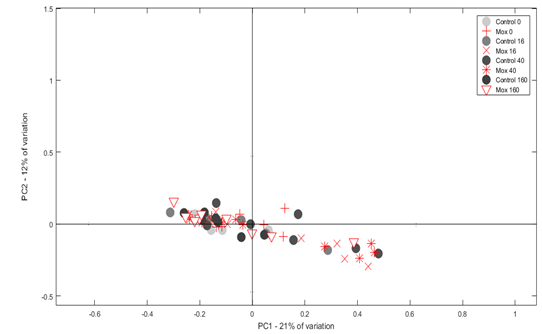

Supplement: Supplementary file 5 — Additional file 5: S5. Scores plot for urine sample metabolites for both moxidectin and control groups at each of the sampling time points, treatment did not alter metabolic profile. [file 42523_2020_56_MOESM5_ESM.png]
